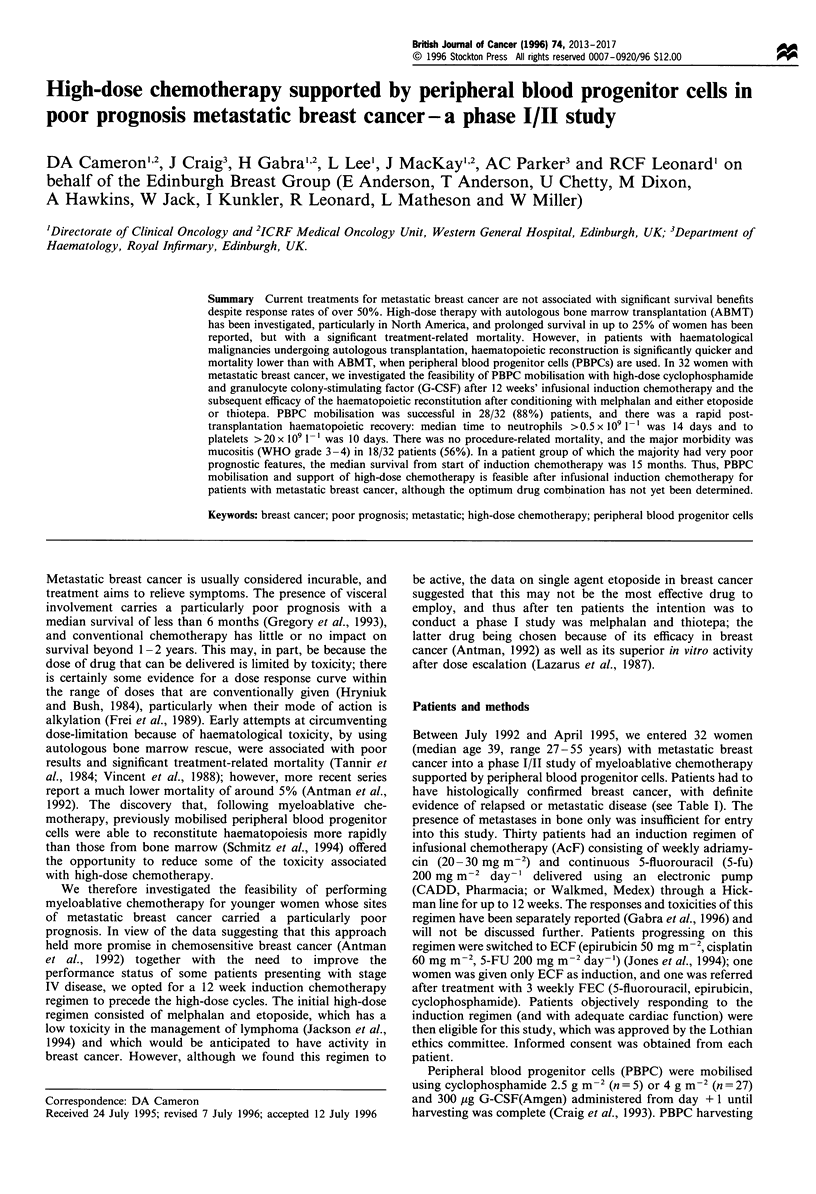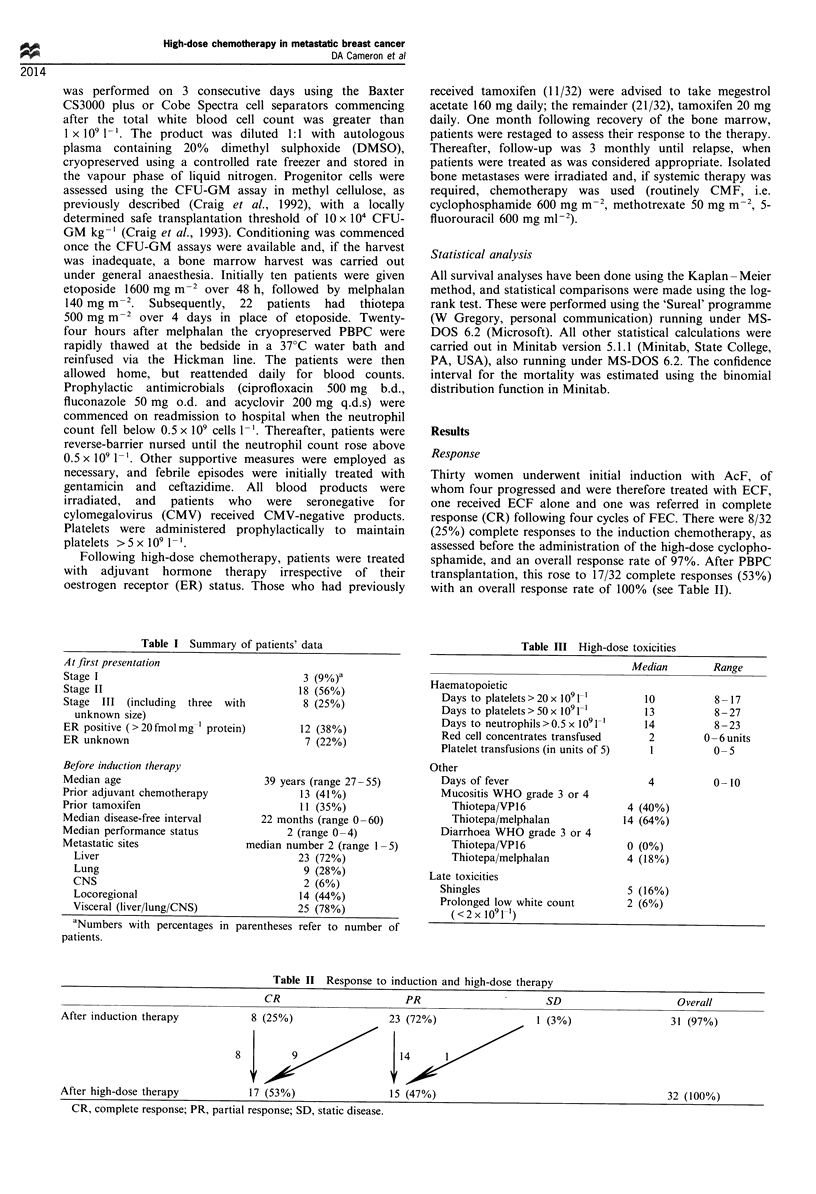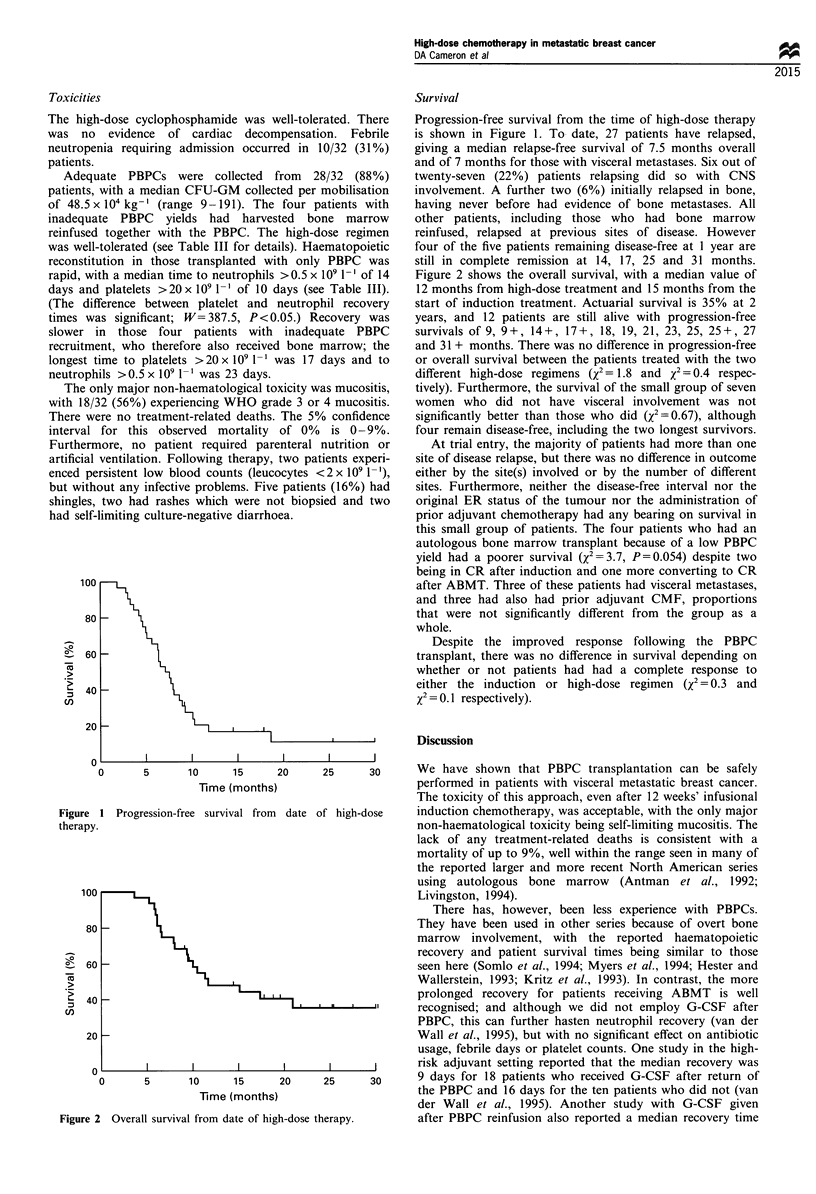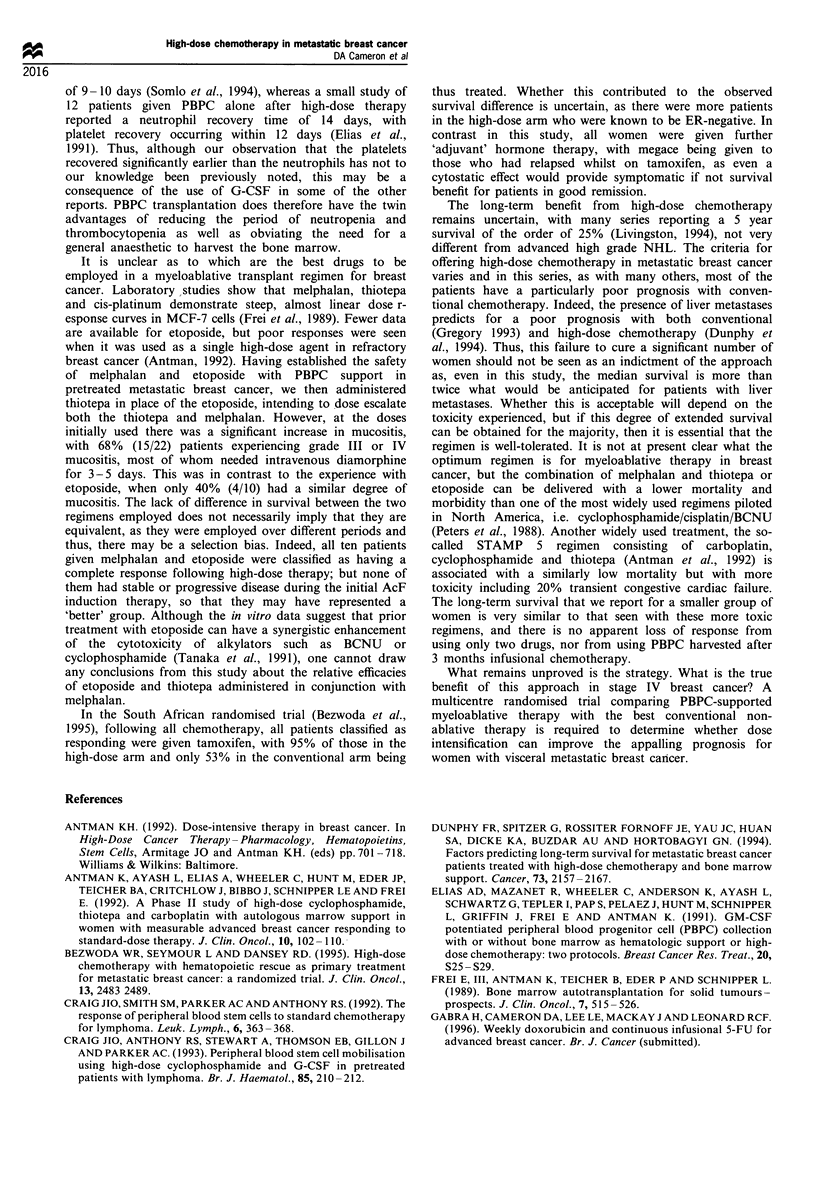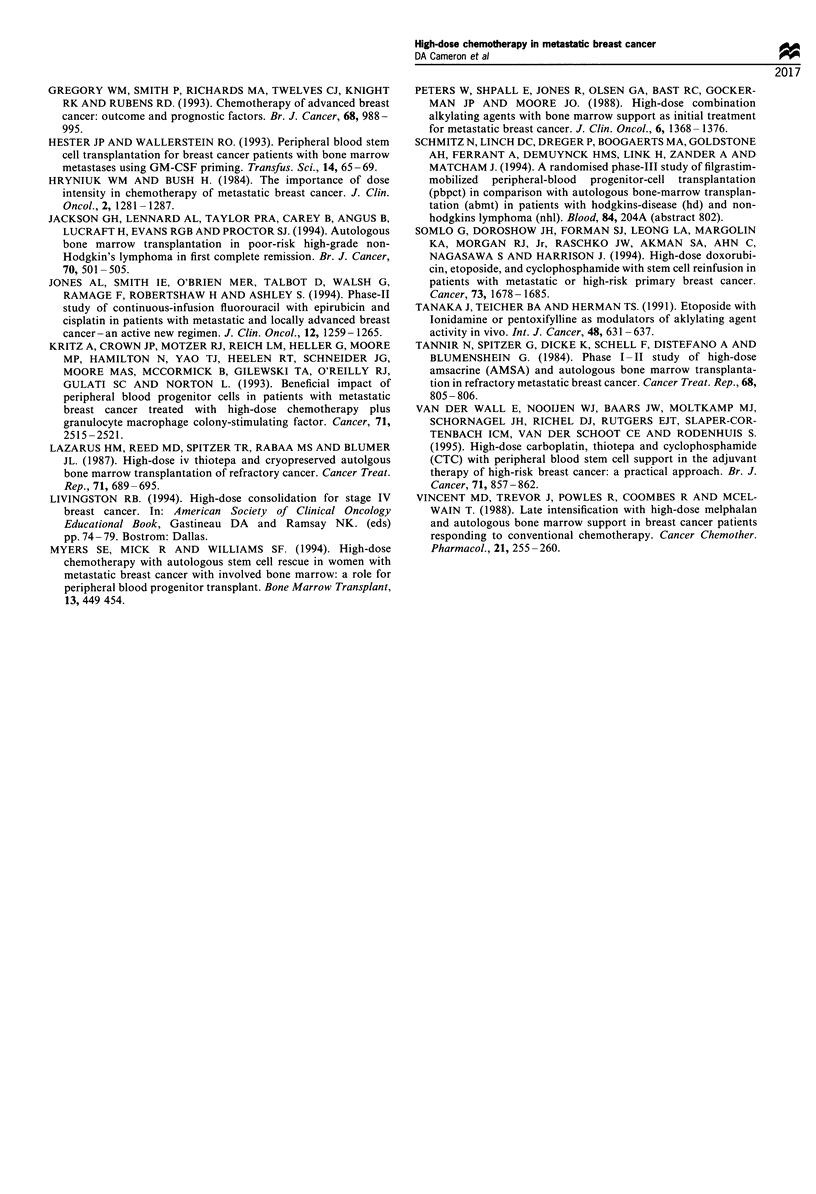# High-dose chemotherapy supported by peripheral blood progenitor cells in poor prognosis metastatic breast cancer--phase I/II study. Edinburgh Breast Group.

**DOI:** 10.1038/bjc.1996.669

**Published:** 1996-12

**Authors:** D. A. Cameron, J. Craig, H. Gabra, L. Lee, J. MacKay, A. C. Parker, R. C. Leonard, E. Anderson, T. Anderson, U. Chetty, M. Dixon, A. Hawkins, W. Jack, I. Kunkler, R. Leonard, L. Matheson, W. Miller

**Affiliations:** Western General Hospital, Edinburgh, UK.

## Abstract

Current treatments for metastatic breast cancer are not associated with significant survival benefits despite response rates of over 50%. High-dose therapy with autologous bone marrow transplantation (ABMT) has been investigated, particularly in North America, and prolonged survival in up to 25% of women has been reported, but with a significant treatment-related mortality. However, in patients with haematological malignancies undergoing autologous transplantation, haematopoietic reconstruction is significantly quicker and mortality lower than with ABMT, when peripheral blood progenitor cells (PBPCs) are used. In 32 women with metastatic breast cancer, we investigated the feasibility of PBPC mobilisation with high-dose cyclophosphamide and granulocyte colony-stimulating factor (G-CSF) after 12 weeks' infusional induction chemotherapy and the subsequent efficacy of the haematopoietic reconstitution after conditioning with melphalan and either etoposide or thiotepa. PBPC mobilisation was successful in 28/32 (88%) patients, and there was a rapid post-transplantation haematopoietic recovery: median time to neutrophils > 0.5 x 10(9) l-1 was 14 days and to platelets > 20 x 10(9) l-1 was 10 days. There was no procedure-related mortality, and the major morbidity was mucositis (WHO grade 3-4) in 18/32 patients (56%). In a patient group of which the majority had very poor prognostic features, the median survival from start of induction chemotherapy was 15 months. Thus, PBPC mobilisation and support of high-dose chemotherapy is feasible after infusional induction chemotherapy for patients with metastatic breast cancer, although the optimum drug combination has not yet been determined.


British Journal of Cancer (1996) 74, 2013-2017
© 1996 Stockton Press All rights reserved 0007-0920/96 $12.00

### High-dose chemotherapy supported by peripheral blood progenitor cells in poor prognosis metastatic breast cancer - a phase I/II study

DA Cameron[1,2], J Craig[3], H Gabra[1,2], L Lee[1], J MacKay[1,2], AC Parker[3] and RCF Leonard[1] on behalf of the Edinburgh Breast Group (E Anderson, T Anderson, U Chetty, M Dixon, A Hawkins, W Jack, I Kunkler, R Leonard, L Matheson and W Miller)

*[1]Directorate of Clinical Oncology and [2]ICRF Medical Oncology Unit, Western General Hospital, Edinburgh, UK; [3]Department of Haematology, Royal Infirmary, Edinburgh, UK.*

**Summary** Current treatments for metastatic breast cancer are not associated with significant survival benefits despite response rates of over 50%. High-dose therapy with autologous bone marrow transplantation (ABMT) has been investigated, particularly in North America, and prolonged survival in up to 25% of women has been reported, but with a significant treatment-related mortality. However, in patients with haematological malignancies undergoing autologous transplantation, haematopoietic reconstruction is significantly quicker and mortality lower than with ABMT, when peripheral blood progenitor cells (PBPCs) are used. In 32 women with metastatic breast cancer, we investigated the feasibility of PBPC mobilisation with high-dose cyclophosphamide and granulocyte colony-stimulating factor (G-CSF) after 12 weeks' infusional induction chemotherapy and the subsequent efficacy of the haematopoietic reconstitution after conditioning with melphalan and either etoposide or thiotepa. PBPC mobilisation was successful in 28/32 (88%) patients, and there was a rapid post-transplantation haematopoietic recovery: median time to neutrophils $>0.5\times10^9\ l^{-1}$ was 14 days and to platelets $>20\times10^9\ l^{-1}$ was 10 days. There was no procedure-related mortality, and the major morbidity was mucositis (WHO grade 3-4) in 18/32 patients (56%). In a patient group of which the majority had very poor prognostic features, the median survival from start of induction chemotherapy was 15 months. Thus, PBPC mobilisation and support of high-dose chemotherapy is feasible after infusional induction chemotherapy for patients with metastatic breast cancer, although the optimum drug combination has not yet been determined.

**Keywords:** breast cancer; poor prognosis; metastatic; high-dose chemotherapy; peripheral blood progenitor cells

Metastatic breast cancer is usually considered incurable, and treatment aims to relieve symptoms. The presence of visceral involvement carries a particularly poor prognosis with a median survival of less than 6 months (Gregory *et al.*, 1993), and conventional chemotherapy has little or no impact on survival beyond 1-2 years. This may, in part, be because the dose of drug that can be delivered is limited by toxicity; there is certainly some evidence for a dose response curve within the range of doses that are conventionally given (Hryniuk and Bush, 1984), particularly when their mode of action is alkylation (Frei *et al.*, 1989). Early attempts at circumventing dose-limitation because of haematological toxicity, by using autologous bone marrow rescue, were associated with poor results and significant treatment-related mortality (Tannir *et al.*, 1984; Vincent *et al.*, 1988); however, more recent series report a much lower mortality of around 5% (Antman *et al.*, 1992). The discovery that, following myeloablative chemotherapy, previously mobilised peripheral blood progenitor cells were able to reconstitute haematopoiesis more rapidly than those from bone marrow (Schmitz *et al.*, 1994) offered the opportunity to reduce some of the toxicity associated with high-dose chemotherapy.

We therefore investigated the feasibility of performing myeloablative chemotherapy for younger women whose sites of metastatic breast cancer carried a particularly poor prognosis. In view of the data suggesting that this approach held more promise in chemosensitive breast cancer (Antman *et al.*, 1992) together with the need to improve the performance status of some patients presenting with stage IV disease, we opted for a 12 week induction chemotherapy regimen to precede the high-dose cycles. The initial high-dose regimen consisted of melphalan and etoposide, which has a low toxicity in the management of lymphoma (Jackson *et al.*, 1994) and which would be anticipated to have activity in breast cancer. However, although we found this regimen to be active, the data on single agent etoposide in breast cancer suggested that this may not be the most effective drug to employ, and thus after ten patients the intention was to conduct a phase I study was melphalan and thiotepa; the latter drug being chosen because of its efficacy in breast cancer (Antman, 1992) as well as its superior *in vitro* activity after dose escalation (Lazarus *et al.*, 1987).

Correspondence: DA Cameron
Received 24 July 1995; revised 7 July 1996; accepted 12 July 1996

### Patients and methods

Between July 1992 and April 1995, we entered 32 women (median age 39, range 27-55 years) with metastatic breast cancer into a phase I/II study of myeloablative chemotherapy supported by peripheral blood progenitor cells. Patients had to have histologically confirmed breast cancer, with definite evidence of relapsed or metastatic disease (see Table I). The presence of metastases in bone only was insufficient for entry into this study. Thirty patients had an induction regimen of infusional chemotherapy (AcF) consisting of weekly adriamycin (20-30 mg $m^{-2}$) and continuous 5-fluorouracil (5-fu) 200 mg $m^{-2}$ $day^{-1}$ delivered using an electronic pump (CADD, Pharmacia; or Walkmed, Medex) through a Hickman line for up to 12 weeks. The responses and toxicities of this regimen have been separately reported (Gabra *et al.*, 1996) and will not be discussed further. Patients progressing on this regimen were switched to ECF (epirubicin 50 mg $m^{-2}$, cisplatin 60 mg $m^{-2}$, 5-FU 200 mg $m^{-2}$ $day^{-1}$) (Jones *et al.*, 1994); one women was given only ECF as induction, and one was referred after treatment with 3 weekly FEC (5-fluorouracil, epirubicin, cyclophosphamide). Patients objectively responding to the induction regimen (and with adequate cardiac function) were then eligible for this study, which was approved by the Lothian ethics committee. Informed consent was obtained from each patient.

Peripheral blood progenitor cells (PBPC) were mobilised using cyclophosphamide 2.5 g $m^{-2}$ ($n=5$) or 4 g $m^{-2}$ ($n=27$) and 300 $\mu$g G-CSF(Amgen) administered from day +1 until harvesting was complete (Craig *et al.*, 1993). PBPC harvesting was performed on 3 consecutive days using the Baxter CS3000 plus or Cobe Spectra cell separators commencing after the total white blood cell count was greater than $1 \times 10^9\ l^{-1}$. The product was diluted 1:1 with autologous plasma containing 20% dimethyl sulphoxide (DMSO), cryopreserved using a controlled rate freezer and stored in the vapour phase of liquid nitrogen. Progenitor cells were assessed using the CFU-GM assay in methyl cellulose, as previously described (Craig *et al.*, 1992), with a locally determined safe transplantation threshold of $10 \times 10^4$ CFU-GM $kg^{-1}$ (Craig *et al.*, 1993). Conditioning was commenced once the CFU-GM assays were available and, if the harvest was inadequate, a bone marrow harvest was carried out under general anaesthesia. Initially ten patients were given etoposide 1600 mg $m^{-2}$ over 48 h, followed by melphalan 140 mg $m^{-2}$. Subsequently, 22 patients had thiotepa 500 mg $m^{-2}$ over 4 days in place of etoposide. Twenty-four hours after melphalan the cryopreserved PBPC were rapidly thawed at the bedside in a 37°C water bath and reinfused via the Hickman line. The patients were then allowed home, but reattended daily for blood counts. Prophylactic antimicrobials (ciprofloxacin 500 mg b.d., fluconazole 50 mg o.d. and acyclovir 200 mg q.d.s) were commenced on readmission to hospital when the neutrophil count fell below $0.5 \times 10^9$ cells $l^{-1}$. Thereafter, patients were reverse-barrier nursed until the neutrophil count rose above $0.5 \times 10^9\ l^{-1}$. Other supportive measures were employed as necessary, and febrile episodes were initially treated with gentamicin and ceftazidime. All blood products were irradiated, and patients who were seronegative for cylomegalovirus (CMV) received CMV-negative products. Platelets were administered prophylactically to maintain platelets $> 5 \times 10^9\ l^{-1}$.

Following high-dose chemotherapy, patients were treated with adjuvant hormone therapy irrespective of their oestrogen receptor (ER) status. Those who had previously received tamoxifen (11/32) were advised to take megestrol acetate 160 mg daily; the remainder (21/32), tamoxifen 20 mg daily. One month following recovery of the bone marrow, patients were restaged to assess their response to the therapy. Thereafter, follow-up was 3 monthly until relapse, when patients were treated as was considered appropriate. Isolated bone metastases were irradiated and, if systemic therapy was required, chemotherapy was used (routinely CMF, i.e. cyclophosphamide 600 mg $m^{-2}$, methotrexate 50 mg $m^{-2}$, 5-fluorouracil 600 mg $ml^{-2}$).

*Statistical analysis*

All survival analyses have been done using the Kaplan–Meier method, and statistical comparisons were made using the log-rank test. These were performed using the 'Sureal' programme (W Gregory, personal communication) running under MS-DOS 6.2 (Microsoft). All other statistical calculations were carried out in Minitab version 5.1.1 (Minitab, State College, PA, USA), also running under MS-DOS 6.2. The confidence interval for the mortality was estimated using the binomial distribution function in Minitab.

### Results

*Response*

Thirty women underwent initial induction with AcF, of whom four progressed and were therefore treated with ECF, one received ECF alone and one was referred in complete response (CR) following four cycles of FEC. There were 8/32 (25%) complete responses to the induction chemotherapy, as assessed before the administration of the high-dose cyclophosphamide, and an overall response rate of 97%. After PBPC transplantation, this rose to 17/32 complete responses (53%) with an overall response rate of 100% (see Table II).

**Table I** Summary of patients' data

| | |
|---|---|
| *At first presentation* | |
| Stage I | 3 (9%)[a] |
| Stage II | 18 (56%) |
| Stage III (including three with unknown size) | 8 (25%) |
| ER positive (> 20 fmol $mg^{-1}$ protein) | 12 (38%) |
| ER unknown | 7 (22%) |
| *Before induction therapy* | |
| Median age | 39 years (range 27–55) |
| Prior adjuvant chemotherapy | 13 (41%) |
| Prior tamoxifen | 11 (35%) |
| Median disease-free interval | 22 months (range 0–60) |
| Median performance status | 2 (range 0–4) |
| Metastatic sites | median number 2 (range 1–5) |
| Liver | 23 (72%) |
| Lung | 9 (28%) |
| CNS | 2 (6%) |
| Locoregional | 14 (44%) |
| Visceral (liver/lung/CNS) | 25 (78%) |

[a]Numbers with percentages in parentheses refer to number of patients.

**Table III** High-dose toxicities

| | *Median* | *Range* |
|---|---|---|
| Haematopoietic | | |
| Days to platelets > $20 \times 10^9\ l^{-1}$ | 10 | 8–17 |
| Days to platelets > $50 \times 10^9\ l^{-1}$ | 13 | 8–27 |
| Days to neutrophils > $0.5 \times 10^9\ l^{-1}$ | 14 | 8–23 |
| Red cell concentrates transfused | 2 | 0–6 units |
| Platelet transfusions (in units of 5) | 1 | 0–5 |
| Other | | |
| Days of fever | 4 | 0–10 |
| Mucositis WHO grade 3 or 4 | | |
| Thiotepa/VP16 | 4 (40%) | |
| Thiotepa/melphalan | 14 (64%) | |
| Diarrhoea WHO grade 3 or 4 | | |
| Thiotepa/VP16 | 0 (0%) | |
| Thiotepa/melphalan | 4 (18%) | |
| Late toxicities | | |
| Shingles | 5 (16%) | |
| Prolonged low white count (< $2 \times 10^9\ l^{-1}$) | 2 (6%) | |

**Table II** Response to induction and high-dose therapy

| | *CR* | *PR* | *SD* | *Overall* |
|---|---|---|---|---|
| After induction therapy | 8 (25%) | 23 (72%) | 1 (3%) | 31 (97%) |
| | ↓ 8 (CR→CR); ↙ 9 (PR→CR) | ↓ 14 (PR→PR); ↙ 1 (SD→PR) | | |
| After high-dose therapy | 17 (53%) | 15 (47%) | | 32 (100%) |

CR, complete response; PR, partial response; SD, static disease.

*Toxicities*

The high-dose cyclophosphamide was well-tolerated. There was no evidence of cardiac decompensation. Febrile neutropenia requiring admission occurred in 10/32 (31%) patients.

Adequate PBPCs were collected from 28/32 (88%) patients, with a median CFU-GM collected per mobilisation of $48.5 \times 10^4$ $kg^{-1}$ (range 9–191). The four patients with inadequate PBPC yields had harvested bone marrow reinfused together with the PBPC. The high-dose regimen was well-tolerated (see Table III for details). Haematopoietic reconstitution in those transplanted with only PBPC was rapid, with a median time to neutrophils $>0.5 \times 10^9$ $l^{-1}$ of 14 days and platelets $>20 \times 10^9$ $l^{-1}$ of 10 days (see Table III). (The difference between platelet and neutrophil recovery times was significant; $W = 387.5$, $P < 0.05$.) Recovery was slower in those four patients with inadequate PBPC recruitment, who therefore also received bone marrow; the longest time to platelets $>20 \times 10^9$ $l^{-1}$ was 17 days and to neutrophils $>0.5 \times 10^9$ $l^{-1}$ was 23 days.

The only major non-haematological toxicity was mucositis, with 18/32 (56%) experiencing WHO grade 3 or 4 mucositis. There were no treatment-related deaths. The 5% confidence interval for this observed mortality of 0% is 0–9%. Furthermore, no patient required parenteral nutrition or artificial ventilation. Following therapy, two patients experienced persistent low blood counts (leucocytes $<2 \times 10^9$ $l^{-1}$), but without any infective problems. Five patients (16%) had shingles, two had rashes which were not biopsied and two had self-limiting culture-negative diarrhoea.

**Figure 1** Progression-free survival from date of high-dose therapy.

**Figure 2** Overall survival from date of high-dose therapy.

*Survival*

Progression-free survival from the time of high-dose therapy is shown in Figure 1. To date, 27 patients have relapsed, giving a median relapse-free survival of 7.5 months overall and of 7 months for those with visceral metastases. Six out of twenty-seven (22%) patients relapsing did so with CNS involvement. A further two (6%) initially relapsed in bone, having never before had evidence of bone metastases. All other patients, including those who had bone marrow reinfused, relapsed at previous sites of disease. However four of the five patients remaining disease-free at 1 year are still in complete remission at 14, 17, 25 and 31 months. Figure 2 shows the overall survival, with a median value of 12 months from high-dose treatment and 15 months from the start of induction treatment. Actuarial survival is 35% at 2 years, and 12 patients are still alive with progression-free survivals of 9, 9+, 14+, 17+, 18, 19, 21, 23, 25, 25+, 27 and 31+ months. There was no difference in progression-free or overall survival between the patients treated with the two different high-dose regimens ($\chi^2 = 1.8$ and $\chi^2 = 0.4$ respectively). Furthermore, the survival of the small group of seven women who did not have visceral involvement was not significantly better than those who did ($\chi^2 = 0.67$), although four remain disease-free, including the two longest survivors.

At trial entry, the majority of patients had more than one site of disease relapse, but there was no difference in outcome either by the site(s) involved or by the number of different sites. Furthermore, neither the disease-free interval nor the original ER status of the tumour nor the administration of prior adjuvant chemotherapy had any bearing on survival in this small group of patients. The four patients who had an autologous bone marrow transplant because of a low PBPC yield had a poorer survival ($\chi^2 = 3.7$, $P = 0.054$) despite two being in CR after induction and one more converting to CR after ABMT. Three of these patients had visceral metastases, and three had also had prior adjuvant CMF, proportions that were not significantly different from the group as a whole.

Despite the improved response following the PBPC transplant, there was no difference in survival depending on whether or not patients had had a complete response to either the induction or high-dose regimen ($\chi^2 = 0.3$ and $\chi^2 = 0.1$ respectively).

### Discussion

We have shown that PBPC transplantation can be safely performed in patients with visceral metastatic breast cancer. The toxicity of this approach, even after 12 weeks' infusional induction chemotherapy, was acceptable, with the only major non-haematological toxicity being self-limiting mucositis. The lack of any treatment-related deaths is consistent with a mortality of up to 9%, well within the range seen in many of the reported larger and more recent North American series using autologous bone marrow (Antman *et al.*, 1992; Livingston, 1994).

There has, however, been less experience with PBPCs. They have been used in other series because of overt bone marrow involvement, with the reported haematopoietic recovery and patient survival times being similar to those seen here (Somlo *et al.*, 1994; Myers *et al.*, 1994; Hester and Wallerstein, 1993; Kritz *et al.*, 1993). In contrast, the more prolonged recovery for patients receiving ABMT is well recognised; and although we did not employ G-CSF after PBPC, this can further hasten neutrophil recovery (van der Wall *et al.*, 1995), but with no significant effect on antibiotic usage, febrile days or platelet counts. One study in the high-risk adjuvant setting reported that the median recovery was 9 days for 18 patients who received G-CSF after return of the PBPC and 16 days for the ten patients who did not (van der Wall *et al.*, 1995). Another study with G-CSF given after PBPC reinfusion also reported a median recovery time of 9–10 days (Somlo *et al.*, 1994), whereas a small study of 12 patients given PBPC alone after high-dose therapy reported a neutrophil recovery time of 14 days, with platelet recovery occurring within 12 days (Elias *et al.*, 1991). Thus, although our observation that the platelets recovered significantly earlier than the neutrophils has not to our knowledge been previously noted, this may be a consequence of the use of G-CSF in some of the other reports. PBPC transplantation does therefore have the twin advantages of reducing the period of neutropenia and thrombocytopenia as well as obviating the need for a general anaesthetic to harvest the bone marrow.

It is unclear as to which are the best drugs to be employed in a myeloablative transplant regimen for breast cancer. Laboratory studies show that melphalan, thiotepa and cis-platinum demonstrate steep, almost linear dose r-esponse curves in MCF-7 cells (Frei *et al.*, 1989). Fewer data are available for etoposide, but poor responses were seen when it was used as a single high-dose agent in refractory breast cancer (Antman, 1992). Having established the safety of melphalan and etoposide with PBPC support in pretreated metastatic breast cancer, we then administered thiotepa in place of the etoposide, intending to dose escalate both the thiotepa and melphalan. However, at the doses initially used there was a significant increase in mucositis, with 68% (15/22) patients experiencing grade III or IV mucositis, most of whom needed intravenous diamorphine for 3–5 days. This was in contrast to the experience with etoposide, when only 40% (4/10) had a similar degree of mucositis. The lack of difference in survival between the two regimens employed does not necessarily imply that they are equivalent, as they were employed over different periods and thus, there may be a selection bias. Indeed, all ten patients given melphalan and etoposide were classified as having a complete response following high-dose therapy; but none of them had stable or progressive disease during the initial AcF induction therapy, so that they may have represented a 'better' group. Although the *in vitro* data suggest that prior treatment with etoposide can have a synergistic enhancement of the cytotoxicity of alkylators such as BCNU or cyclophosphamide (Tanaka *et al.*, 1991), one cannot draw any conclusions from this study about the relative efficacies of etoposide and thiotepa administered in conjunction with melphalan.

In the South African randomised trial (Bezwoda *et al.*, 1995), following all chemotherapy, all patients classified as responding were given tamoxifen, with 95% of those in the high-dose arm and only 53% in the conventional arm being thus treated. Whether this contributed to the observed survival difference is uncertain, as there were more patients in the high-dose arm who were known to be ER-negative. In contrast in this study, all women were given further 'adjuvant' hormone therapy, with megace being given to those who had relapsed whilst on tamoxifen, as even a cytostatic effect would provide symptomatic if not survival benefit for patients in good remission.

The long-term benefit from high-dose chemotherapy remains uncertain, with many series reporting a 5 year survival of the order of 25% (Livingston, 1994), not very different from advanced high grade NHL. The criteria for offering high-dose chemotherapy in metastatic breast cancer varies and in this series, as with many others, most of the patients have a particularly poor prognosis with conventional chemotherapy. Indeed, the presence of liver metastases predicts for a poor prognosis with both conventional (Gregory 1993) and high-dose chemotherapy (Dunphy *et al.*, 1994). Thus, this failure to cure a significant number of women should not be seen as an indictment of the approach as, even in this study, the median survival is more than twice what would be anticipated for patients with liver metastases. Whether this is acceptable will depend on the toxicity experienced, but if this degree of extended survival can be obtained for the majority, then it is essential that the regimen is well-tolerated. It is not at present clear what the optimum regimen is for myeloablative therapy in breast cancer, but the combination of melphalan and thiotepa or etoposide can be delivered with a lower mortality and morbidity than one of the most widely used regimens piloted in North America, i.e. cyclophosphamide/cisplatin/BCNU (Peters *et al.*, 1988). Another widely used treatment, the so-called STAMP 5 regimen consisting of carboplatin, cyclophosphamide and thiotepa (Antman *et al.*, 1992) is associated with a similarly low mortality but with more toxicity including 20% transient congestive cardiac failure. The long-term survival that we report for a smaller group of women is very similar to that seen with these more toxic regimens, and there is no apparent loss of response from using only two drugs, nor from using PBPC harvested after 3 months infusional chemotherapy.

What remains unproved is the strategy. What is the true benefit of this approach in stage IV breast cancer? A multicentre randomised trial comparing PBPC-supported myeloablative therapy with the best conventional non-ablative therapy is required to determine whether dose intensification can improve the appalling prognosis for women with visceral metastatic breast cancer.

### References

ANTMAN KH. (1992). Dose-intensive therapy in breast cancer. In *High-Dose Cancer Therapy–Pharmacology, Hematopoietins, Stem Cells*, Armitage JO and Antman KH. (eds) pp. 701–718. Williams & Wilkins: Baltimore.

ANTMAN K, AYASH L, ELIAS A, WHEELER C, HUNT M, EDER JP, TEICHER BA, CRITCHLOW J, BIBBO J, SCHNIPPER LE AND FREI E. (1992). A Phase II study of high-dose cyclophosphamide, thiotepa and carboplatin with autologous marrow support in women with measurable advanced breast cancer responding to standard-dose therapy. *J. Clin. Oncol.*, **10**, 102–110.

BEZWODA WR, SEYMOUR L AND DANSEY RD. (1995). High-dose chemotherapy with hematopoietic rescue as primary treatment for metastatic breast cancer: a randomized trial. *J. Clin. Oncol.*, **13**, 2483 2489.

CRAIG JIO, SMITH SM, PARKER AC AND ANTHONY RS. (1992). The response of peripheral blood stem cells to standard chemotherapy for lymphoma. *Leuk. Lymph.*, **6**, 363–368.

CRAIG JIO, ANTHONY RS, STEWART A, THOMSON EB, GILLON J AND PARKER AC. (1993). Peripheral blood stem cell mobilisation using high-dose cyclophosphamide and G-CSF in pretreated patients with lymphoma. *Br. J. Haematol.*, **85**, 210–212.

DUNPHY FR, SPITZER G, ROSSITER FORNOFF JE, YAU JC, HUAN SA, DICKE KA, BUZDAR AU AND HORTOBAGYI GN. (1994). Factors predicting long-term survival for metastatic breast cancer patients treated with high-dose chemotherapy and bone marrow support. *Cancer*, **73**, 2157–2167.

ELIAS AD, MAZANET R, WHEELER C, ANDERSON K, AYASH L, SCHWARTZ G, TEPLER I, PAP S, PELAEZ J, HUNT M, SCHNIPPER L, GRIFFIN J, FREI E AND ANTMAN K. (1991). GM-CSF potentiated peripheral blood progenitor cell (PBPC) collection with or without bone marrow as hematologic support or high-dose chemotherapy: two protocols. *Breast Cancer Res. Treat.*, **20**, S25–S29.

FREI E, III, ANTMAN K, TEICHER B, EDER P AND SCHNIPPER L. (1989). Bone marrow autotransplantation for solid tumours–prospects. *J. Clin. Oncol.*, **7**, 515–526.

GABRA H, CAMERON DA, LEE LE, MACKAY J AND LEONARD RCF. (1996). Weekly doxorubicin and continuous infusional 5-FU for advanced breast cancer. *Br. J. Cancer* (submitted).

GREGORY WM, SMITH P, RICHARDS MA, TWELVES CJ, KNIGHT RK AND RUBENS RD. (1993). Chemotherapy of advanced breast cancer: outcome and prognostic factors. *Br. J. Cancer*, **68,** 988–995.

HESTER JP AND WALLERSTEIN RO. (1993). Peripheral blood stem cell transplantation for breast cancer patients with bone marrow metastases using GM-CSF priming. *Transfus. Sci.*, **14,** 65–69.

HRYNIUK WM AND BUSH H. (1984). The importance of dose intensity in chemotherapy of metastatic breast cancer. *J. Clin. Oncol.*, **2,** 1281–1287.

JACKSON GH, LENNARD AL, TAYLOR PRA, CAREY B, ANGUS B, LUCRAFT H, EVANS RGB AND PROCTOR SJ. (1994). Autologous bone marrow transplantation in poor-risk high-grade non-Hodgkin's lymphoma in first complete remission. *Br. J. Cancer*, **70,** 501–505.

JONES AL, SMITH IE, O'BRIEN MER, TALBOT D, WALSH G, RAMAGE F, ROBERTSHAW H AND ASHLEY S. (1994). Phase-II study of continuous-infusion fluorouracil with epirubicin and cisplatin in patients with metastatic and locally advanced breast cancer – an active new regimen. *J. Clin. Oncol.*, **12,** 1259–1265.

KRITZ A, CROWN JP, MOTZER RJ, REICH LM, HELLER G, MOORE MP, HAMILTON N, YAO TJ, HEELEN RT, SCHNEIDER JG, MOORE MAS, MCCORMICK B, GILEWSKI TA, O'REILLY RJ, GULATI SC AND NORTON L. (1993). Beneficial impact of peripheral blood progenitor cells in patients with metastatic breast cancer treated with high-dose chemotherapy plus granulocyte macrophage colony-stimulating factor. *Cancer*, **71,** 2515–2521.

LAZARUS HM, REED MD, SPITZER TR, RABAA MS AND BLUMER JL. (1987). High-dose iv thiotepa and cryopreserved autolgous bone marrow transplantation of refractory cancer. *Cancer Treat. Rep.*, **71,** 689–695.

LIVINGSTON RB. (1994). High-dose consolidation for stage IV breast cancer. In: *American Society of Clinical Oncology Educational Book*, Gastineau DA and Ramsay NK. (eds) pp. 74–79. Bostrom: Dallas.

MYERS SE, MICK R AND WILLIAMS SF. (1994). High-dose chemotherapy with autologous stem cell rescue in women with metastatic breast cancer with involved bone marrow: a role for peripheral blood progenitor transplant. *Bone Marrow Transplant*, **13,** 449 454.

PETERS W, SHPALL E, JONES R, OLSEN GA, BAST RC, GOCKERMAN JP AND MOORE JO. (1988). High-dose combination alkylating agents with bone marrow support as initial treatment for metastatic breast cancer. *J. Clin. Oncol.*, **6,** 1368–1376.

SCHMITZ N, LINCH DC, DREGER P, BOOGAERTS MA, GOLDSTONE AH, FERRANT A, DEMUYNCK HMS, LINK H, ZANDER A AND MATCHAM J. (1994). A randomised phase-III study of filgrastim-mobilized peripheral-blood progenitor-cell transplantation (pbpct) in comparison with autologous bone-marrow transplantation (abmt) in patients with hodgkins-disease (hd) and non-hodgkins lymphoma (nhl). *Blood*, **84,** 204A (abstract 802).

SOMLO G, DOROSHOW JH, FORMAN SJ, LEONG LA, MARGOLIN KA, MORGAN RJ, Jr, RASCHKO JW, AKMAN SA, AHN C, NAGASAWA S AND HARRISON J. (1994). High-dose doxorubicin, etoposide, and cyclophosphamide with stem cell reinfusion in patients with metastatic or high-risk primary breast cancer. *Cancer*, **73,** 1678–1685.

TANAKA J, TEICHER BA AND HERMAN TS. (1991). Etoposide with Ionidamine or pentoxifylline as modulators of aklylating agent activity in vivo. *Int. J. Cancer*, **48,** 631–637.

TANNIR N, SPITZER G, DICKE K, SCHELL F, DISTEFANO A AND BLUMENSHEIN G. (1984). Phase I–II study of high-dose amsacrine (AMSA) and autologous bone marrow transplantation in refractory metastatic breast cancer. *Cancer Treat. Rep.*, **68,** 805–806.

VAN DER WALL E, NOOIJEN WJ, BAARS JW, MOLTKAMP MJ, SCHORNAGEL JH, RICHEL DJ, RUTGERS EJT, SLAPER-CORTENBACH ICM, VAN DER SCHOOT CE AND RODENHUIS S. (1995). High-dose carboplatin, thiotepa and cyclophosphamide (CTC) with peripheral blood stem cell support in the adjuvant therapy of high-risk breast cancer: a practical approach. *Br. J. Cancer*, **71,** 857–862.

VINCENT MD, TREVOR J, POWLES R, COOMBES R AND MCELWAIN T. (1988). Late intensification with high-dose melphalan and autologous bone marrow support in breast cancer patients responding to conventional chemotherapy. *Cancer Chemother. Pharmacol.*, **21,** 255–260.